# Estimating the burden of acute gastrointestinal illness due to *Giardia, Cryptosporidium, Campylobacter, E. coli* O157 and norovirus associated with private wells and small water systems in Canada

**DOI:** 10.1017/S0950268815002071

**Published:** 2015-11-13

**Authors:** H. M. MURPHY, M. K. THOMAS, P. J. SCHMIDT, D. T. MEDEIROS, S. McFADYEN, K. D. M. PINTAR

**Affiliations:** 1 Centre for Food-borne, Environmental and Zoonotic Infectious Diseases, Public Health Agency of Canada, Guelph, ON, Canada; 2 Department of Civil and Environmental Engineering, University of Waterloo, Waterloo, ON, Canada; 3 Water and Air Quality Bureau, Healthy Environments and Consumer Safety Branch, Health Canada, Ottawa, ON, Canada

**Keywords:** Water (quality), waterborne illness, burden, small drinking water systems, private wells

## Abstract

Waterborne illness related to the consumption of contaminated or inadequately treated water is a global public health concern. Although the magnitude of drinking water-related illnesses in developed countries is lower than that observed in developing regions of the world, drinking water is still responsible for a proportion of all cases of acute gastrointestinal illness (AGI) in Canada. The estimated burden of endemic AGI in Canada is 20·5 million cases annually – this estimate accounts for under-reporting and under-diagnosis. About 4 million of these cases are domestically acquired and foodborne, yet the proportion of waterborne cases is unknown. There is evidence that individuals served by private systems and small community systems may be more at risk of waterborne illness than those served by municipal drinking water systems in Canada. However, little is known regarding the contribution of these systems to the overall drinking water-related AGI burden in Canada. Private water supplies serve an estimated 12% of the Canadian population, or ~4·1 million people. An estimated 1·4 million (4·1%) people in Canada are served by small groundwater (2·6%) and surface water (1·5%) supplies. The objective of this research is to estimate the number of AGI cases attributable to water consumption from these supplies in Canada using a quantitative microbial risk assessment (QMRA) approach. This provides a framework for others to develop burden of waterborne illness estimates for small water supplies. A multi-pathogen QMRA of *Giardia, Cryptosporidium, Campylobacter, E. coli* O157 and norovirus, chosen as index waterborne pathogens, for various source water and treatment combinations was performed. It is estimated that 103 230 AGI cases per year are due to the presence of these five pathogens in drinking water from private and small community water systems in Canada. In addition to providing a mechanism to assess the potential burden of AGI attributed to small systems and private well water in Canada, this research supports the use of QMRA as an effective source attribution tool when there is a lack of randomized controlled trial data to evaluate the public health risk of an exposure source. QMRA is also a powerful tool for identifying existing knowledge gaps on the national scale to inform future surveillance and research efforts.

## INTRODUCTION

The magnitude and sources of waterborne (enteric) illness in Canada are not well-defined. Enteric illness is largely under-reported, and existing national and provincial surveillance systems for enteric illness do not distinguish between infections caused by food, animal contact, person-to-person, environmental, or drinking water transmission. The Public Health Agency of Canada estimates that there are roughly 20·5 million AGI cases each year (0·6 cases/person per year) [1]. Of the overall burden, we estimate that 4 million cases are foodborne (and acquired domestically) [1], while the remaining cases are attributed to water, animal contact, and person-to-person transmission.

Private water supplies (households) serve an estimated 12% of the Canadian population, or ~4·1 million people [2]. Eleven percent of Canadians are supplied by a private (unregulated) groundwater source, and 1% use a private surface water source (e.g. a spring, lake, river or dugout) [2]. The responsibility of managing and maintaining the quality of these water supplies falls to their owners [3]. Health Canada recommends that households on private wells have their water tested by a laboratory 2–3 times per year [4]; however, only 27% of households on private water supplies had their water tested in 2011 [2].

An estimated 1·7 million (4·9%) Canadians are served by small community groundwater (3·1%) and surface water (1·8%) supplies [2, 5]. For this study, a small supply is defined as a system serving <1000 people. Private and small community water systems may be more at risk for human illness than municipally operated systems in Canada [6–9]. In a review of Canadian waterborne outbreaks between 1974 and 2001, two-thirds of the outbreaks occurred at either private or semi-private systems [10]. *Campylobacter* spp., *Cryptosporidium* spp., *Giardia* spp., and *E. coli* O157 were responsible for the majority (~58%) of these outbreaks, which is consistent with European trends [11]. In addition, Schuster *et al.* [10] found that Norwalk-like viruses and rotavirus were responsible for 9·3% (14/150) of Canadian outbreaks where causative organisms were identified. This is consistent with findings in the United States [12], where 6% of waterborne outbreaks were due to norovirus.

There is some evidence to suggest that private well and small water system users may be at increased risk of AGI; however, the magnitude of this risk had not been quantified in Canada. The objective of the work presented herein is to estimate the number of AGI cases associated with *Giardia, Cryptosporidium, Campylobacter, E. coli* O157 and norovirus associated with the consumption of water from private and small community systems in Canada using a quantitative microbial risk assessment (QMRA) approach. This study is part of a comprehensive approach to quantify and attribute AGI in Canada to various sources to inform policy, research and surveillance efforts [13].

## MATERIALS AND METHODS

### Model framework

**Fig. 1.**
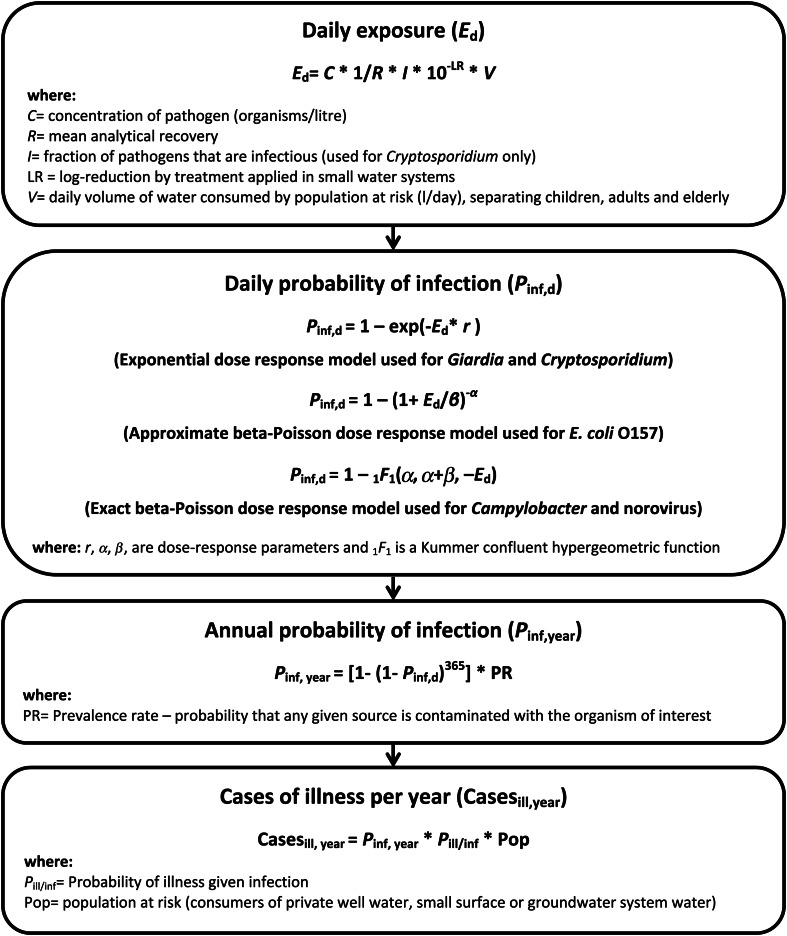
Schematic diagram of QMRA model used to estimate the disease burden of *Giardia, Cryptosporidium, Campylobacter, E. coli* O157 and norovirus from the consumption of water from private wells and small water systems serving <1000 people (adapted from Haas *et al.* [14]; Howard *et al.* [15]).

A multi-pathogen QMRA was developed using *Giardia, Cryptosporidium, Campylobacter, E. coli* O157 and norovirus as these are the pathogens most commonly associated with enteric drinking water outbreaks in Canada. Five stochastic models were developed to estimate the number of AGI cases caused by each pathogen, separated into three types of drinking water supplies: private groundwater wells, small groundwater supplies (serving <1000 individuals), and small surface water supplies (serving <1000 individuals). The QMRA methodology and subsequent equations applied were adapted from Haas *et al.* [14] and Howard *et al.* [15] (Fig. 1). The daily probability of infection in the present study represents the probability of being infected following exposure to a water supply that is contaminated with the pathogen of interest. The annual probability of infection represents the probability of infection over a 1-year period accounting for the possibility of exposure to a water supply that is positive for pathogens (prevalence rate). In this analysis, the conversion from probability of illness to cases of illness assumed a maximum of one illness per year.

QMRA was used because no randomized control trials have been performed on small community supplies or private wells [16]. To capture the uncertainty and variability associated with the estimates, inputs were described using probability distributions. The minimum, maximum, and most likely values for the variables were developed (using PERT and Uniform distributions, or raw data fit to parametric distributions such as Lognormal or Weibull) [17]. The final estimates [reported as mean with 90% probability interval (PI) around the mean] were generated using Monte Carlo simulation [10 000 iterations using @Risk (Palisade Corp., USA)]. Sensitivity analyses were performed by examining the Spearman correlation coefficients (*r*_*s*_).

### Model inputs

#### Population at risk

Using the 2011 Canadian population (34 342 800) [18], and data on the proportion of Canadians that consumed water from private and small system groundwater and surface water supplies [2], the approximate population on each of these water supplies was estimated: 1 068 830 individuals on small municipal groundwater systems, 614 128 individuals on small surface water systems, 4 138 080 individuals on private wells, and 22 246 976 individuals on large municipal systems. The proportion of Canadians that report exclusively consuming bottled water and the proportion of households that reportedly treat their water at the intake to their home and state that this is to treat for bacteria [2] were excluded (Table 1). The proportion of children (⩽10 years), adults (11–64 years) and elderly ⩾65 years) were estimated [18] to generate age-specific incidence rates (Table 2).

**Table 1.** Estimation of the population at risk that consumes water from private wells and small system supplies in Canada

Population category	Private wells	Small GW systems	Small SW systems	Total
A. Total population	4 138 080	1 068 830	614 128	5 821 038
B. Proportion of people that drink bottled water exclusively (27%*, 22%† of A)	1 117 282	235 143	135 108	1 487 533
C. Proportion of tap water households that treat their water at intake to home [34%*, 5%† of (A – B)]	1 027 071	41 684	23 951	1 092 706
D. Estimated proportion of households that treat for bacteria (31%*, 32%† of C)	318 392	13 339	7664	339 395
E. Distribution of population at risk: Uniform[(A – B – D); (A – B)]	(2 702 406–3 020 798)	(820 348–833 687)	(471 356–479 020)	

GW, Groundwater; SW, surface water.

* Applies to private wells.

† Applies to small GW and small SW systems.

**Table 2.** Population served by private wells and small system water supplies in Canada, and water consumption distribution inputs by age group (children, adults, elderly)

Population category*	Estimated population ranges (minimum–maximum)			Water consumption distributions (l/day) lognormal, mean (s.d.)
	Private wells	Small GW systems	Small SW systems	
Children (⩽10 years)	462 111–516 556	140 280–142 560	80 602–81 912	1·207 (0·632)
Adults (11–64 years)	1 756 564–1 963 519	533 226–541 897	306 381–311 363	1·500 (0·924)
Elderly (⩾65 years)	483 731–540 723	146 842–149 230	84 373–85 745	1·260 (0·650)

GW, Groundwater; SW, surface water.

* Age categories in the 2011 census (Statistics Canada [5]) were ⩽10 years for children (17·1%); 15–64 years for adults (65%); ⩾65 years for the elderly (17·9%). No adjustments were made to reconcile these census categories with the water consumption categories.

#### Water consumption

Water consumption data collected from a Canadian community survey were used to develop consumption inputs for three age groups: ⩽10 years (*n* = 132), 11–64 years (*n* = 1636) and ⩾65 years (*n* = 331) (raw data from [19]), and fit to a lognormal distribution [20].

#### Canadian groundwater and surface water pathogen occurrence and concentration data

All inputs for both pathogen prevalence rates (PR) and concentration (*C*) in groundwater [21] and surface water were derived from the published literature (Tables 3, 4; Supplementary Tables S1, S2). Data were obtained from those studies that examined pathogen occurrence under ‘non-outbreak’ conditions. Canadian pathogen data were included where available. In the absence of Canadian data, US studies were used. Where Canadian or US data were unavailable, international studies were used. It was assumed that reported non-detects or zero values from the literature were zero.

**Table 3.** Groundwater pathogen inputs selected for use in the private well and small groundwater system QMRA models for *Giardia, Cryptosporidium, Campylobacter, E. coli* O157 and norovirus

Model parameter	Distribution	Rationale	Reference
*Giardia*			
Prevalence rate	Point estimate (1·34%)	1·34% of wells were positive with *Giardia* in US study; nearly all other US/Canada studies reported no detection of *Giardia* in well water	Hancock *et al.* [48]
Concentration (cysts/l)	Lognormal* (0·01, 0·025)	Data were obtained from one paper, which collected 253 samples from 149 wells across the United States in different geographical locations (only study of this magnitude in North America). Reported median substituted for arithmetic mean	Hancock *et al.* [48]
Recovery	Point estimate (47%)	Middle of range reported by Hancock *et al.* [48]	Hancock *et al.* [48]
*Cryptosporidium*			
Prevalence rate	PERT‡ (0%, 4·7%, 6·2%)	0% is the lowest occurrence from Isaac-Renton *et al.* [62]; 4·7% is the prevalence from Hancock *et al.* [48] because it is the largest published study; 6·2% is based on pooled US/Canada positive-well results (i.e. 12/193)	Hancock *et al.* [48]; Isaac-Renton *et al.* [62]; Betancourt & Rose, [45]; Budu-Amoako *et al.* [46, 47]
% Infectious	Point estimate (50%)	It is assumed that not all *Cryptosporidium* in groundwater will be of infectious origin based on findings for surface water (Table 2). It is estimated that the proportion in groundwater may be higher than surface water due to septic tank inputs. Therefore, 50% was chosen, guided by informal expert advice
Concentration (oocysts/l)	Lognormal* (0·02, 0·09)	Data selected from one study that collected 253 samples from 149 wells across the United States in different geographical locations (only study of this magnitude in North America). Results from other studies fit within the range reported by Hancock *et al.* [48], except for two outlier data points reported by Budo-Amoako *et al.* [46, 47] of 7·2 and 8·83 oocysts/l in agriculturally intensive areas of Prince Edward Island. Reported median substituted for arithmetic mean	Hancock *et al.* [48]; Isaac-Renton *et al.* [62]; Betancourt & Rose, [45]; Budu-Amoako *et al.* [46, 47]
Recovery	Point estimate (17·5%)	Middle of range reported by Hancock *et al.* [48]	Hancock *et al.* [48]
*Campylobacter*			
Prevalence rate	Point estimate (1·37%)	US/Canada studies pooled to develop point estimate (i.e. 5/364)	Borchardt *et al.* [41]; St. Pierre *et al.* [63]
Concentration (MPN/l)	Exponential§ (0·28, truncated at 4)	Concentration distribution obtained directly from New Zealand QMRA study because North American concentration data for wells were not available	Close *et al.* [64]
Recovery	Point estimate (100%)	Conservatively high value	
*E. coli* O157			
Prevalence rate	Point estimate (1·9%)	US/Canadian studies pooled to develop point estimate (i.e. 7/371)	Borchardt *et al.* [41]; Won *et al.* [65]
Concentration (c.f.u./l)	Uniform† (0·001, 0·01)	Concentration distribution obtained from only US/Canadian paper that reports concentration data for *E. coli* O157 in groundwater	Won *et al.* [65]
Recovery	Point estimate (100%)	Conservatively high value	
Norovirus			
Prevalence rate	PERT‡ (0·95%, 3·52%, 6·25%)	Contamination rates taken from pooled US/Canadian results of all norovirus samples. Min = lowest (sample) % occurrence [66]; most likely = % of samples from pooled studies for norovirus (excluding Borchardt *et al.* [67] GII data); max = highest sample % occurrence [68]	Abbazadegan *et al.* [66]; Borchardt *et al.* [68]; Locas *et al.* [69]; Hunt *et al.* [70]; Borchardt *et al.* [67]; Allen [71]
Concentration (gc/l)	Weibull|| (0·6267, 23·72)	Fit to raw untreated groundwater data for norovirus GI obtained from Dr Borchardt (2010 data)	Unpublished data
Recovery	Point estimate (100%)	Conservatively high value	

c.f.u., Colony-forming units; gc, genomic copies; MPN, most probable number; QMRA, quantitative microbial risk assessment.

* Lognormal distribution (arithmetic mean, standard deviation).

†Uniform distribution (minimum, maximum).

‡ PERT distribution (minimum, most likely, maximum).

§ Exponential distribution (scale parameter).

|| Weibull distribution (shape parameter, scale parameter).

**Table 4.** Surface water pathogen inputs selected for use in the small systems QMRA models for *Giardia, Cryptosporidium, Campylobacter, E. coli* O157 and norovirus

Model parameter	Distribution	Rationale	Reference
*Giardia*			
Prevalence rate	PERT* (0%, 19%, 42%)	Selected range published by Wilkes *et al.* [72] because study captured range of prevalence in different seasons.	Wilkes *et al.* [72]
Concentration (cysts/l)	Lognormal† (0·176, 2·14)	Health Canada data from multiple source waters were fit to lognormal distribution. Reported median substituted for arithmetic mean	Unpublished Health Canada data; Wilkes *et al.* [73]; Payment *et al.* [74]
Recovery	−	Health Canada data were adjusted for recovery	Unpublished Health Canada data
*Cryptosporidium*			
Prevalence rate	PERT* (0%, 17%, 72%)	Selected range published by Wilkes *et al.* [72], reflecting prevalence across different seasons. Range compares to values published by a second Canadian surface water study [75]	Wilkes *et al.* [72]; Ruecker *et al.* [75]
% Infectious	PERT* (0%, 4·1%, 8%)	Based on % samples positive *for C. hominis, C. parvum*. Min, 0%; most likely (mean of all studies, 8%, 1·6%, 2·7%); max (8%) from Pintar *et al*. [76]	Pintar *et al.* [76]; Ruecker *et al.* [75]; Wilkes *et al.* [39]
Concentration (oocyst/l)	Lognormal† (0·0857, 0·4269)	Health Canada data from multiple source waters were fit to lognormal distribution.	Unpublished Health Canada data; Wilkes *et al.* [73]; Payment *et al.* [74]; Ruecker *et al.* [77]
Recovery	−	Health Canada data were adjusted for recovery	Unpublished Health Canada data
*Campylobacter*			
Prevalence rate	PERT* (0%, 25%, 57·8%)	0% is the lowest seasonal prevalence rate; 25% is the overall prevalence rate from Wilkes *et al*. [73]; 57·8% is the largest seasonal prevalence rate	St Pierre *et al.* [63]; Wilkes *et al.* [72, 73];
Concentration (MPN/l)	Lognormal† (3·04, 255)	Data from one Canadian study [63] and MPN data from FoodNet Canada were fit to a lognormal distribution	St Pierre *et al.* [63]; FoodNet Canada [78]
Recovery	Point estimate (100%)	Conservatively high value	
*E. coli* O157			
Prevalence rate	PERT* (0%, 1%, 2·3%)	Low, most likely and high values selected from pooled literature	Johnson *et al.* [79]; Wilkes *et al.* [72, 73]; Jokinen *et al.* [80]
Concentration (c.f.u./l)	Uniform‡ (0·001, 0·01)	Used same values as Won *et al.* [65] paper (groundwater)	Won *et al.* [65]
Recovery	Point estimate (100%)	Conservatively high value	
Norovirus			
Prevalence rate	PERT* (0%, 6·3%, 10%)	Data from freshwater recreational waters in Europe and Canadian rivers	Wyn-Jones *et al.* [40]; Wilkes *et al.* [39]
Concentration (gc/l)	PERT* (1, 10, 400)	Only North American study that reported concentrations in gc/l	Corsi *et al.* [81]
Recovery	Point estimate (100%)	Conservatively high value	

c.f.u., Colony-forming units; gc, genomic copies; MPN, most probable number; QMRA, quantitative microbial risk assessment.

* PERT distribution (minimum, most likely, maximum) – expert judgement informed the choice of minimum, mode and maximum values when the literature were sparse (for example, a minimum value of 0% was included based on our understanding that samples could be negative for this pathogen).

† Lognormal distribution (arithmetic mean, standard deviation).

‡ Uniform distribution (minimum, maximum).

For small groundwater and surface water systems, the level of treatment applied (log reduction) was considered based on the treatment systems currently in place in Canada (Fig. 1). Using data from the Survey of Drinking Water Plants [5], 19 treatment types were developed (Supplementary Table S3: Treatment types). These treatment types were further combined into the following five categories.

1. No treatment.
2. Membrane filtration (micro- or ultra-filtration) with chemical disinfection (with/without other treatment, but no UV disinfection).
3. Media filtration (with/without other treatment, but no UV or membrane filtration).
4. Chemical disinfection (ozone, chlorine, chlorine dioxide, or chloramines) with/without coagulation/ flocculation/sedimentation, but no filtration or UV disinfection.
5. UV and chemical disinfection (with/without other treatment).

Specific log removal/inactivation ranges were developed using the minimum and maximum levels of treatment that could be achieved for each pathogen and each of the original 19 treatment categories and source water types (ground *vs*. surface). These values were developed from literature references compiled by Health Canada for various treatment types for five reference pathogens: *Giardia, Cryptosporidium*, rotavirus (used as a proxy for norovirus treatment in this QMRA), *Campylobacter and E. coli* (based on studies used for the Health Canada QMRA model) [34]. As no single virus has all the characteristics of an ideal reference virus, this risk assessment incorporates the key characteristics of rotavirus, with CT values based on hepatitis A virus (HAV) and poliovirus [United States Environmental Protection Agency (USEPA, 1999)] as the best currently available disinfection information for enteric viruses commonly found in surface water and groundwater sources (Health Canada, 2011). For each of the five treatment categories above, the log-reduction ranges were combined and PERT log removal distributions were developed such that each source water type, treatment category, and pathogen has a unique distribution. Mode values are based upon population-weighted fitting (sampling from population size/treatment level combinations for each pathogen/treatment combination) (Supplementary Table S5). Applying a PERT distribution is less sensitive to the extremes of the distribution than a triangular distribution and more sensitive to the most likely value (mode) [17].

Previously published dose-response models for each of the five pathogens were obtained from the peer-reviewed literature (Table 5). The morbidity factor (*P*_ill/inf_) for each organism is presented in Table 5. These values were based on an in-depth literature review conducted by USEPA [22].

**Table 5.** Dose-response functions and morbidity values selected for the *Giardia, Cryptosporidium, Campylobacter, E. coli* O157 and norovirus models

Pathogen	Dose response and morbidity	Reference
*Giardia*	Exponential, *r* = 0·0199 *P*_ill/inf_ = Uniform distribution (0·2–0·7)	Rose *et al.* [82] USEPA [22]
*Cryptosporidium*	Exponential, *r* = 0·018 *P*_ill/inf_ = Uniform distribution (0·2–0·7)	Messner *et al.* [83] USEPA [22]
*Campylobacter*	Exact beta-Poisson (*α* = 0·1453, *β* = 8·007) *P*_ill/inf_ = Uniform distribution (0·1–0·6)	Schmidt *et al.* [84] USEPA [22]
*E. coli* O157	Approx. beta-Poisson (*α* = 0·4, *β* = 37·6) *P*_ill/inf_ = Uniform distribution (0·2–0·6)	Median of 10 000 iterations by Teunis *et al.* [85]; Bielaszewska *et al.* [86] USEPA, [22]
Norovirus*	Exact beta-Poisson (*α* = 0·04, *β* = 0·055) *P*_ill/inf_ = Uniform distribution (0·3–0·8)	Teunis *et al.* [87] USEPA [21]

* It was assumed that the entire population lacks immunity to norovirus.

## RESULTS

Estimated daily and annual probabilities of infection, numbers of cases of infection/year and the corresponding cases of illness/year for all five pathogens for all three water supplies are presented in Tables 6, 7, and Supplementary Tables S6–S8.

**Table 6.** Estimated total number of domestically acquired Canadian cases of *Giardia, Cryptosporidium, Campylobacter, E. coli* O157 and norovirus attributable to private wells, small groundwater systems and small surface water systems

Pathogen	Total estimated Canadian cases* (90% PI)	Projected illnesses attributable to private wells (90% PI)	Projected illnesses attributable to small GW systems (90% PI)	Projected illnesses attributable to small SW systems (90% PI)	% of total cases related to small/ private systems†
*Giardia*	108 507 (70 532–158 740)	1207 (2–7136)	121 (0–619)	2288 (6–12 120)	3·33%
*Cryptosporidium*	25 318 (14 331–45 955)	11 398 (238–45 141)	1639 (27–7108)	317 (1–1310)	52·7%
*Campylobacter*	213 479 (144 288–308 837)	9273 (1180–19 980)	378 (45–818)	513 (1–1433)	4·76%
*E. coli* O157	16 913 (6968–29 668)	637 (124–1528)	28 (5–72)	1 (0–4)	3·94%
Norovirus	3 379 990 (3 002 927–3 778 461)	55 558 (24 323–95 709)	10 869 (2211–22 736)	9003 (1790–18 930)	2·23%
Total projected cases		78 073 (38 466–128 109)	13 035 (3416–25 698)	12 122 (2974–26 274)	

GW, Groundwater; SW, surface water; PI, Probability interval.

* Data from Public Health Agency of Canada foodborne illness estimates [1]

† Sum of projected illnesses attributable to private wells and small systems divided by total estimated Canadian cases.

**Table 7.** Comparison of AGI incidence rates by water source type and treatment system category

Treatment category	Private wells			Small groundwater systems			Small surface water systems		
	Annual AGI cases	Population served	Incidence*	Annual AGI cases	Population served	Incidence*	Annual AGI cases	Population served	Incidence*
No treatment	78 073	2 861 602	0·027	2948	108 014	0·027	597	6066	0·098
Membrane filtration	−	−	−	365	45 952	0·008	788	46 202	0·017
Media filtration	−	−	−	1991	166 698	0·012	3370	160 789	0·021
Chemical disinfection	−	−	−	7720	485 244	0·016	7295	197 716	0·037
UV and chemical disinfection	−	−	−	10	21 111	0·0005	73	64 417	0·011

AGI, Acute gastrointestinal illness.

* Incidence rate = cases/p-yr.

### Private wells

QMRA models estimated a total of 78 073 cases of illness/year (90% PI 38 466-128 109) resulting from consumption of water containing *Giardia, Cryptosporidium, Campylobacter, E. coli* O157 and norovirus from untreated private wells in Canada (Supplementary Table S6). This corresponds to ~0·027 AGI cases/person-year (p-yr) for those consuming water from these supplies. Norovirus is estimated to be responsible for 71·2% of symptomatic cases (*n* = 55 558), followed by *Campylobacter* (*n* = 9273, 11·9%), *Cryptosporidium* (*n* = 11398, 14·6%), *Giardia* (1207, 1·53%) *and E. coli* O157 (637, 0·82%).

### Small groundwater systems

The small groundwater systems models predicted that 13 035 cases of illness/year (90% PI 3416-25 698) are attributable to *Giardia, Cryptosporidium, Campylobacter, E. coli* O157 and norovirus associated with the consumption of water from small groundwater supplies serving <1000 people (Supplementary Table S7). This is an incidence of ~0·016 AGI cases/p-yr for those consuming water from these supplies. Similar to private wells, norovirus was responsible for the majority of cases (*n*  = 10869, 83·4%) followed by *Cryptosporidium* (*n* = 1639, 12·6%), *Campylobacter* (*n* = 378, 2·90%), *Giardia* (*n* = 121, 0·93%) and *E. coli* O157 (*n* = 28, 0·21%).

### Small surface water systems

It is estimated that 12 122 cases of illness/year (90% PI 2974-26 274) are attributable to the consumption of water from small Canadian surface water systems (serving <1000 people) as a result of *Giardia, Cryptosporidium, Campylobacter, E. coli* O157 and norovirus (Supplementary Table S8). This corresponds to an incidence of 0·026 AGI cases/p-yr for those consuming water from these supplies. Norovirus is responsible for the majority of cases of illness (*n* = 9003, 74·3%), followed by *Giardia* (*n* = 2288, 18·9%), *Campylobacter* (*n* = 513, 4·23%), *Cryptosporidium* (*n* = 317, 2·62%) and *E. coli* O157 (*n* = 1, 0·008%).

### Waterborne AGI

In Canada, it is estimated that there are 20·5 million AGI cases that occur annually (0·6 cases/p-yr), after accounting for under-diagnosis and under-reporting [1]. This includes both domestically acquired and travel-acquired AGI. Of the total 20·5 million, an estimated 4 million (20%) domestically acquired cases are attributed to food [1]. The current study estimates that ~103 230 cases (0·003/p-yr), or 0·51%, of the total are attributed to the consumption of water from private wells and small water systems (Table 6).

When comparing cumulative projected AGI incidence rates from all reference pathogens by system type and treatment category, those served by surface water systems with no treatment (0·098 cases/p-yr) or only one treatment barrier, such as chemical disinfection (0·037 cases/p-yr) are more at risk than those served by groundwater systems or private wells (Table 7). Incidence rates for those served by untreated groundwater or private wells were the same (0·027 cases/p-yr) and were slightly lower than for those served by groundwater systems treated by chemical disinfection (0·016 cases/p-yr), membrane filtration (0·008 cases/p-yr) or media filtration (0·012 cases/p-yr). The lowest incidence rates were estimated for individuals served by surface water or groundwater sources with both UV and chemical disinfection (0·0005–0·001 cases/p-yr).

### Sensitivity analysis

Sensitivity analyses were performed to determine model input contributions to each model (Supplementary Table S4). Pathogen concentration was predictably correlated with estimated numbers of illnesses, appearing in the top three factors in all pathogen models with the exception of norovirus (*r* = 0·442–0·866), and was the most highly correlated input in both protozoan models. The lack of correlation for norovirus is due to the estimated high exposures for a small fraction of private wells and small systems; for which, the corresponding annual probability of infection is nearly 100%. The prevalence rate was significantly correlated with outputs from the *Cryptosporidium, E. coli* O157 and norovirus models (*r* = 0·267–0·755). Daily adult water consumption was a significant input in both the *Giardia* (*r* = 0·187) and *Campylobacter* (*r* = 0·195) models.

## DISCUSSION

This study is part of a larger effort to quantify and attribute AGI in Canada to various sources, including drinking water. The objective of the work presented herein is to estimate the number of AGI cases caused by *Giardia, Cryptosporidium, Campylobacter, E. coli* O157 and norovirus attributable to the consumption of water from private wells and small community systems in Canada using a QMRA approach.

In Canada, the majority of drinking water systems are small systems. There are significant challenges faced by these systems, which have been identified nationally and internationally as a priority in the drinking water treatment and public health communities. The World Health Organization (WHO) has identified management of small community drinking water supplies as a critical issue for sustainable development and health [23]. The Office of the Inspector General within the USEPA also conducted an evaluation and concluded that effort and resources are needed to help small drinking water systems overcome the challenges to delivering safe drinking water [24]. In Canada, federal, provincial and territorial governments have long recognized the importance of supporting small drinking water systems in the delivery of safe water.

Small drinking water systems face the same issues as larger municipal drinking water systems in the provision of safe water. In 2011, the National Collaborating Centre for Environmental Health (NCCEH) published a report that examined the issue of waterborne disease risks in Canadian small drinking water systems. They found a high proportion of waterborne disease outbreaks occurred in small drinking water systems serving <5000 people [25]. While the challenges small systems face are similar to large systems, they are usually operating with fewer people, who have less experience and technical knowledge [26]. Small systems often find it difficult to hire and retain staff with sufficient technical knowledge. In addition, they often have limited time to dedicate to the operation of these systems, as drinking water operators/managers are often responsible for other operations in the community [27]. Distribution system issues exist in small systems too, including inadequate maintenance, high water losses, cross-connections, frequent breaks and slow replacement [25]. Northern communities have unique challenges, due to the presence of permafrost, extreme cold and lack of year-round road networks, as well as difficulty accessing laboratories.

Source protection, adequate treatment and continued monitoring are the tenets of a safe and robust drinking water supply, regardless of size [28]. With small systems, there are more elusive factors associated with the effective operation of a treatment system and delivery of safe water. A recent Canadian study emphasizes the utility of developing water safety plans for small and large water systems and the importance of building community readiness through early engagement [29] to reduce the risk and burden of waterborne illness.

### Risk by drinking water source and treatment category

The combination of source water type (ground *vs.* surface) and level of drinking water treatment influence the microbial and chemical quality of drinking water. This study illustrates that those individuals served by small surface water systems could be more at risk of AGI than those served by private wells or small groundwater systems (in terms of incidence rates, as shown in Table 7). This study also illustrates that those served by untreated surface water supplies could be at greatest risk for waterborne AGI, although only about 6000 Canadians rely on water from this category. The risk of exposure to waterborne pathogens in small untreated surface water supplies has previously been well documented [12]. Conversely, drinking water systems that treat with UV disinfection are estimated to be at much lower risk for AGI, for both ground and surface water sources. This is attributed to the efficacy of UV disinfection for the inactivation of pathogens in water [30–32].

Small water systems typically have fewer treatment barriers in place compared to larger systems and risk is inherently greater [5, 9]. Small surface water supplies that only chlorinate will be more vulnerable to *Cryptosporidium* and *Giardia* as these organisms are highly resistant to chlorine; whereas a system with multiple barriers (e.g. coagulation, flocculation, filtration, disinfection) is more effective at removing these protozoa [33, 34].

This work illustrates the efficacy of UV disinfection at inactivating pathogens, reducing the likelihood of AGI cases attributed to drinking water. UV disinfection is highly effective (>4 log) at inactivating *Giardia, Cryptosporidium, Campylobacter, E. coli* O157 and norovirus [34]. Although membrane filtration is a robust treatment technology, microfiltration has a nominal pore in a range that will allow viruses to pass through. It is not as effective against rotavirus as UV disinfection [34].

This study also illustrates the impact of private well water quality on the overall burden of waterborne illness in Canada. Engaging private homeowners in the important responsibility of maintaining their wells, treating their drinking water supply, and routinely testing their water is a shared responsibility. Public engagement on the issue of safe well water and stewardship continues to be limited by complacency, inconvenience, cost and privacy concerns, identified consistently by those examining stewardship behaviour of private well owners in Newfoundland [35], Ontario [36], and across Canada [37].

### Norovirus

We estimate between 74% (private wells) and 83% (small groundwater systems) of predicted AGI cases are attributed to norovirus. However, the norovirus dose-response relationship and mechanistic interpretation is subject to considerable debate [38] and may need to be revised as improved dose-response information becomes available.

Few published studies have examined the prevalence and concentration of norovirus in Canadian surface water and groundwater systems and this is an identified knowledge gap. The prevalence rate for norovirus in surface waters was based on one Canadian study of rivers [39] and a study of recreational waters in Europe [40]. With respect to groundwater, we recognize that the developed models do not reflect variations in local hydrogeology, which have a significant influence on the transient nature of virus prevalence and concentrations [41].

Norovirus contributes greatly to the total burden of enteric disease in Canada (~3·4 million cases annually) [1], yet is both under-reported and under-diagnosed, and is not routinely included in national surveillance platforms for enteric illness. In this study, it is estimated that 2·23% of all annual, domestically acquired norovirus cases in Canada could be the result of consumption of water from small or private Canadian water supplies. This is similar to what has been reported in other countries; in the United States, 1·5% of reported norovirus outbreaks between 2010 and 2012 were linked to waterborne transmission [42]. Similarly, norovirus was found to be the most common pathogen contributing to waterborne disease in groundwater systems in Norway [43]. Future work to consider the impact of secondary transmission in the epidemiology of waterborne norovirus infections could consider the rate of community cases attributed back to these initial illnesses, given its high infectivity.

### *Cryptosporidium* and *Giardia*

The developed model predictions that 13 354 and 3616 cases of *Cryptosporidium* and *Giardia,* respectively, occur in Canada each year from the consumption of untreated or inadequately treated groundwater or surface water from private wells and small water systems. The disease burden for *Giardia* infections in Canada is high and ranks in the top three reported enteric infections provincially and nationally, with a national incidence rate of 11·43 cases/100 000 people per year [44]. While the cryptosporidiosis disease burden in Canada is lower (1.82 cases/100000 people per year), the number of predicted cases of *Cryptosporidium* is roughly 9 and 14 times greater in private wells and small groundwater supplies, respectively, than the predicted cases of *Giardia* in the same systems.

Occurrence studies show that *Cryptosporidium* oocysts are more prevalent in groundwater sources than *Giardia* cysts [45–48], primarily due to their smaller size, which allows for more rapid downward ingress through overburden layers [49]. Moreover, *Cryptosporidium* oocysts are more resistant to low subsurface temperatures and thus persist in the environment for longer periods of time [50]. In addition, *Cryptosporidium* is more resistant to chemical disinfection than *Giardia* [51]. Many private or small system groundwater sources are actually undocumented GUDI (groundwater under the direct influence of surface water), which explains the reported presence of oocysts/cysts in these waters [52]. In fact, the presence of large diameter pathogens such as *Cryptosporidium* or *Giardia* in groundwater is sometimes used to define a GUDI water source [53].

### Campylobacter

*Campylobacter* is the leading cause of bacterial gastrointestinal illness in Canada, with an incidence of 29·3 cases/100 000 people per year in 2012 [44]. In this study, we estimate that *Campylobacter* is the third leading cause of waterborne AGI in private well and small water system users in Canada. This represents an estimated 4·76% of an estimated 213 000 domestically acquired cases of *Campylobacter* that occur annually in Canada, after accounting for under-reporting and under-diagnosis [1]. A recent source attribution study of campylobacteriosis using a systematic review and meta-analysis framework demonstrated that untreated drinking water is a significant risk factor [54]. Previous Canadian studies have demonstrated that private wells are a risk factor for campylobacteriosis [55].

### *E. coli* O157

The *E. coli* O157 QMRA model estimated that 666 cases were attributable to the consumption of untreated or inadequately treated water from private wells, small surface water or small groundwater supplies. In Canada, the incidence rate for verotoxigenic *E. coli* (VTEC) is 1·81 cases/100 000 people per year based on reported cases [44]. In a review of *E. coli* O157 outbreaks 1982–2002 in the United States, 15% of all outbreak cases were linked to the consumption of drinking water [56]. Similarly, a source attribution study of *E. coli* O157 infections in the United States found that 5% of sporadic infections and 73% of outbreak infections were associated with consumption of untreated water [57]. In Canada, private well water and small system supplies are recognized risks of *E. coli* O157 infection [58, 59].

### Study limitations/model uncertainty

These models rely on data from the literature and some Canadian surveillance systems, and thus are not site-specific and do not reflect the influence of local hydrogeology, as well as temporal and spatial differences specific to local systems. Rather, the models are designed to reflect the range of likely scenarios for small water systems and private wells in Canada. The inputs are designed to encompass both site-to-site variability and uncertainty in the enteric pathogen concentration and prevalence rates of Canadian groundwater and surface water supplies. The models are limited by data availability. Where pathogen data were available, few studies documented pathogen concentrations in the source water; only 10 of 22 North American studies of *Campylobacter, E. coli* O157 and norovirus documented concentrations, of which two reported only zero values/non-detects, and the remaining reported presence/absence results (Supplementary Tables S1, S2). While we attempted to capture the influence of seasonality on input distributions, by including multi-year studies, these data were not always available. The concentration and prevalence rate (occurrence) of pathogens in Canadian source waters, collected over multiple years, are crucial data needed to refine future burden estimates.

Dose-response models continue to be a limitation of all QMRA models [60]. Additionally, the ratios for the probability of illness given infection do not account for variations we would expect in various sub-populations or the dependence of morbidity upon dose [61]. The uncertainty around the infectivity of different strains and genotypes of an organism, as well as the large variability in host/pathogen interactions, contributes to the uncertainty in the outputs. Future knowledge gaps that could be explored include: (1) rigorous reporting of outbreak details (attack rates for different subtypes of organisms), and (2) community aetiology studies to examine the prevalence of asymptomatic carriage of pathogens.

Deriving a burden estimate by focusing on specific (reference) pathogens may under-estimate illness. There is the potential that there are additional AGI cases attributable to other pathogens, such as *Shigella, Salmonella*, and other enteric viruses [10]. The continued development and optimization of (clinical and environmental) pathogen detection methods will help to further inform which pathogens are most often associated with AGI, and refine our estimate. Despite these inherent limitations, the five pathogens that were used are recognized waterborne reference pathogens (for both the WHO and Health Canada drinking water guidelines) and thus, are considered to adequately represent the majority of known microbial risks from these water supplies. Another consideration that was not explored in this analysis but could influence our understanding of risk is the role that socioeconomic factors play in the burden of waterborne disease, both in urban and rural areas.

Finally, it is important to note that this study did not consider waterborne illness from recreational water, or water for other uses such as irrigation, medical uses, or building water systems. Other types of waterborne illness, such as respiratory illnesses, eye, ear or skin infections, and wound infections were also not considered. Future iterations of this work could assess the burden of the broader spectrum of waterborne diseases, to inform the appropriate allocation of resources and public health interventions.

## CONCLUSIONS

Based on QMRA estimates, the consumption of untreated private well water or inadequately treated water from small surface water and groundwater supplies in Canada may be responsible for an estimated 103 230 AGI cases annually (90% PI 52 419–166 286), representing a small proportion of the burden when considering other routes of transmission. By pathogen, waterborne AGI is attributed to norovirus (73%), *Cryptosporidium* (13%), *Campylobacter* (10%), *Giardia* (3·5%), and *E. coli* O157 (0·6%) in this study.

The results indicate that those served by small surface water systems in Canada with no treatment or inadequate treatment (e.g. chemical disinfection only) are more at risk for AGI than those served by untreated or inadequately treated groundwater supplies. Private wells and small water systems on both surface and groundwater systems are potential sources of parasitic, viral and bacterial infections. Previous Canadian studies focusing on waterborne outbreaks have made similar conclusions [9, 10]. The results continue to illustrate the efficacy of water safety interventions, including the combined use of physical removal with UV and chemical disinfection, for small and private systems, regardless of water source, to reduce public health risk and AGI rates.

Small drinking water systems face the same issues as larger systems with respect to producing potable water; however, they typically have fewer resources, experience and technical knowledge. This is a significant challenge when faced with additional operational objectives, such as implementing a multiple barrier approach based on risk assessment and risk management. The key strength of multi-barrier systems is that the limitations or failure of one or more barriers may be compensated by the effective operation of the remaining barriers [88]. A multiple barrier approach requires an understanding of the hazards, an assessment of the potential risks through the entire drinking water system, and resources to implement a management plan to address those risks. New research focusing on the importance of community engagement in the delivery of safe water, and the utility of water safety plans [29] may help to inform next steps for communities relying on small water systems. Engaging private well owners in the stewardship of their wells is an ongoing responsibility shared by private well owners and governments at all levels, and can be facilitated by effective knowledge translation strategies to inform Canadians about known risks and effective treatment technologies to reduce risk.

In addition to providing a mechanism to assess the potential burden of AGI attributed to small systems and private well water in Canada, this research demonstrates that QMRA is an effective source attribution tool when there is a lack of randomized controlled trial data to evaluate the public health risk of an exposure source. QMRA is also a powerful tool for identifying existing knowledge gaps on the national scale to inform future surveillance and research efforts.

## Supplementary material

For supplementary material accompanying this paper visit https://doi.org/10.1017/S0950268815002071. click here to view supplementary material
