# Supplementary material for: Estimating the burden of acute gastrointestinal illness due to Giardia, Cryptosporidium, Campylobacter, E. coli O157 and norovirus associated with private wells and small water systems in Canada
Source: Epidemiol Infect. 2015 Nov 13;144(7):1355–70. doi: 10.1017/S0950268815002071 (PMC4823832; doi:10.1017/S0950268815002071)
Supplement: Supplementary file 1 [file S0950268815002071sup001.doc]

**Supplementary Material**

Table S1. Groundwater pathogen data for *Giardia, Cryptosporidium, Campylobacter, E. coli* O157 and norovirus used to inform inputs into the QMRA models designed to estimate the number of cases of illness attributable to the consumption of private water supplies (includes study location, pathogen detection method, method recovery, total wells and samples positive and concentration data).

| **Pathogen** | **Reference, publication date** | **Study Location** | **Detection Method** | **Method Recovery** | **Total Wells** | **Wells Positive** | **Total Samples** | **Samples Positive** | **Concentration** |
| --- | --- | --- | --- | --- | --- | --- | --- | --- | --- |
| ***Giardia*** | Hancock *et al.* 1998 | United States | USEPA ICR^1^ Protozoan Method;  Contrast Microscopy | 34-60% | 149 | 2 | 253 | nr^7^ | 8 cysts / 100L (mean)  2 cyst/ 100L (median)  25 cysts/ 100L (st.dev)  0.1- 120 cysts/100L (range) |
|  | Isaac- Renton *et al.* 1999 | British Columbia, Canada | USEPA 1623: IMS^2^, Epifluorescence microscopy | nr | nr | 0 | 35 | 0 | nr |
|  | Betancourt & Rose 2005 | Florida, United States | USEPA 1623 | nr | 4 | 1 | 11 | 1 | 0.7cysts/ 100L ± 1 ( average)  <0.2- 1.5 cysts/ 100L (range) |
|  | Budo-Amoako *et al.* 2012a | PEI, Canada | USEPA 1623 | nr | 20 | 0 | 20 | 0 | 0/ 100L |
|  | Budo-Amoako *et al.* 2012b | PEI, Canada | USEPA 1623 | 29.44 to 32.45% (mean 30.88%) | 20 | 0 | 20 | 0 | 0/ 100L |
| ***Cryptosporidium*** | Hancock *et al.* 1998 | United States | USEPA ICR^1^ Protozoan Method;  Contrast Microscopy | 14-21% | 149 | 7 | 253 | nr | 5 oocysts / 100L (mean)  2 oocysts/ 100L (median)  9 oocysts/100L (st. dev)  0.2- 45 oocysts/100L (range) |
|  | Isaac- Renton *et al.* 1999 | British Columbia, Canada | USEPA 1623 | nr | nr | 0 | 35 | 0 | nr |
|  | Betancourt & Rose 2005 | Florida, United States | USEPA 1623 | nr | 4 | 1 | 11 | 2 | 0.3 oocysts/ 100L  0.1-0.53 oocyst /100L (range) |
|  | Budo-Amoako *et al.* 2012a | PEI, Canada | USEPA 1623 | nr | 20 | 1 | 20 | 1 | 8.83 oocyst/ L |
|  | Budo-Amoako *et al.* 2012b | PEI, Canada | USEPA 1623 | 49.22- 55% (mean 51.5%) | 20 | 3 | 20 | 3 | 0.1- 7.2 oocyst/ L ( range) |
| ***Campylobacter*** | Borchardt *et al.* 2003 | Wisconsin, USA | Culture methods | nr | 191 | 0 | 191 | 0 | 0 cfu/4 L |
|  | St. Pierre, 2009 | Quebec, Canada,  (Groundwater wells) | Culture methods, PCR^3^ confirmation | nr | 120 | 0 | 120 | 0 | nr |
|  | St. Pierre, 2009 | Quebec, Canada  (Surface wells) | Culture methods, PCR^3^ confirmation | nr | 53 | 5 | 53 | 5 | nr for wells |
|  | Close *et al.* 2009 | New Zealand | MPN^4^/ PCR^3^ method | nr | 6 | 6 | 135 | 16 | <0.6 to > 3.1 MPN/ L fit to a truncated exponential distribution |
| ***E. coli* O157** | Borchardt *et al.* 2003 | Wisconsin, United States | Culture | nr | 191 | 0 | 191 | 0 | 0/ 4 L |
|  | Won *et al.* 2013 | Ohio, United States | RT-PCR^5^ | nr | 180 | 7 | 180 | 7 | 10^-6^ to 10^-5^ CFU/ mL |
| **Norovirus GI** | Abbaszadegan *et al.* 2003 | United States | RT-PCR^5^ | nr | 317 | 3 | 317 | 3 | nr |
| **GI** | Borchardt *et al.* 2004 | Wisconsin, USA | RT-PCR^5^ | nr | 6 | 3 | 48 | 3 | nr |
| **Non-specific** | Locas *et al.* 2007 | Quebec, Canada | RT-PCR^5^ | nr | 12 | 3 | 113 | 3 | nr |
| **GI**  **GII** | Hunt *et al.* 2010 | Wisconsin, USA | RT-qPCR^6^ | nr | 5  33 | 2  1 | 40  33 | 2  1 | Sample concentrations :  7.3 gc^8^/ L; 13.6 gc/L  77gc/L |
| **GI**  **GII** | Borchardt *et al.* 2012 | Wisconsin, USA | RT-qPCR^6^ | 29% (Lambertini *et al.* 2012) | 14  14 | nr  nr | 1204  1204 | 51  0 | Mean 0.6 gc/L , Max 116gc/L; 95%ile =0 gc/L |
| **GII** | Allen, 2013 | Guelph, Canada | RT-qPCR^6^ | 29% (Lambertini *et al.* 2012) | 22 | 3 | 118 | 3 | Sample concentrations:  3.34 gc/L; 15.16 gc/L; 15.63 gc/L |

^1^ICR- Information Collection Rule

^2^IMS- Immunomagnetic separation

^3^PCR- Polymerase chain reaction

^4^MPN- Most probable number

^5^RT-PCR- Reverse Transcription- Polymerase Chain Reaction

^6^RT-qPCR- Reverse Transcription – Quantitative Polymerase Chain Reaction

^7^nr – not reported

^8^gc- genomic copies

Table S2. Surface water pathogen data for *Giardia, Cryptosporidium, Campylobacter, E. coli* O157 and norovirus used to inform inputs into the QMRA models designed to estimate the number of cases of illness attributable to the consumption of private water supplies (includes study location, pathogen detection method, method recovery, total samples positive and concentration data).

| **Pathogen** | **Reference, publication date** | | **Study Location** | | **Source water type** | | **Detection Method** | | **Method Recovery** | | | **Total Samples** | | **Samples Positive** | | | **% Positive** | | | **Concentration** | | | | | | | |  |
| --- | --- | --- | --- | --- | --- | --- | --- | --- | --- | --- | --- | --- | --- | --- | --- | --- | --- | --- | --- | --- | --- | --- | --- | --- | --- | --- | --- | --- |
|  |  |  |  |  |  |  |  |  |  |  |  |  |  |  |  |  |  |  |  | **Mean** | | **St. Deviation** | | **Median** | | **Range** | |  |
| ***Giardia*** | Unpublished Health Canada (HC) data, 2013 | | Canada-wide; 11 sites | | Rivers and lakes | | USEPA 1623 | | 69% | | | Nr | | nr | | | nr | | | 2.11 cyst/ L ^1^ | | 2.14cyst/ L^1^ | | 0.176 cyst/L^1^ | | 0- 18.9 cyst/L | |  |
|  | Wilkes *et al.* 2009 | | Ontario, Canada | | River and streams | | USEPA 1623 | | nr | | | 514 | | 129 | | | 19-42% (depending on season) | | | nr | | nr | | 0 cyst/L | | Max 3.6 cyst/L | |  |
|  | Wilkes *et al.* 2011 | | Ontario, Canada | | River and streams | | USEPA 1623 | | nr | | | 664 | | 170 | | | 26% | | | 0.055 cyst/L | | 0.207 cyst/L | | nr | | 0-3.6 cyst/L | |  |
|  | Payment *et al.* 2000 | | Quebec, Canada | | St. Laurent River | | Membrane filtration & Immunofluorescence | | nr | | | Nr | | nr | | | 60% | | | 2.08 cyst/L^2^ | | 3.52 cyst/L^2^ | | 0.735 cyst/ L^2^ | |  | |  |
| ***Cryptosporidium*** | | Unpublished HC data,  2013 | | Canada-wide; 11 sites | | Rivers and lakes | | USEPA 1623 | | | 40% | | nr | | nr | | | nr | | 0.118  oocyst/L^1^ | | 0.144 oocyst/L^1^ | 0.0633 oocyst/L^1^ | | | 0-0.55 oocyst/L | |  |
|  | | Wilkes *et al.* 2009 | | Ontario, Canada | | River and streams | | USEPA 1623 | | | nr | | 514 | | 290 | | | 17-72% depending on season | | nr | | nr | 0 | | | Max= 8 oocyst/L | |  |
|  | | Ruecker *et al.* 2007 | | Ontario, Canada | | River and creeks | | USEPA 1623 | | | nr | | 113 | | 86 | | | 77% | | 0.28 oocyst/L^2^ | | 0.26 oocysts/L^2^ | 0.18 oocysts/ L^2^ | | | 0.04-1.5 oocysts/L | |  |
|  | | Ruecker *et al.* 2012 | | Ontario, Canada | | River and creeks | | USEPA 1623 | | | nr | | 317 | | 674 | | | 47% (20-72% depending on site) ;  1.6% samples human infectious | | nr | | nr | nr | | | 0.033-1.7 oocysts/L | |  |
|  | | Wilkes *et al.* 2011 | | Ontario, Canada | | River and streams | | USEPA 1623 | | | nr | | 664 | | 306 | | | 46% | | 0.25 oocyst/L | | 2.453 oocyst/L | nr | | | 0-  61.33 oocyst/L | |  |
|  | | Pintar *et al.* 2012 | | Ontario, Canada | | River | |  | | | nr | | nr | | nr | | | 3% samples C. parvum  5% C. hominis | | nr | | nr | nr | | | nr | |  |
|  | | Payment *et al.* 2000 | | Quebec, Canada | | St. Laurent River | | Membrane filtration & Immunofluorescence | | | nr | | nr | | nr | | | 40% | | 0.41 oocyst/L^2^ | | 0.625 oocyst/L^2^ | 0.21 oocyst/ L^2^ | | |  | |  |
|  | | Wilkes *et al.* 2013 | | Canada | | Agriculturally impacted streams | | USEPA 1623 | | | nr | | nr | | nr | | | 46%-63% (18/657; 2.7% human infectious) | | nr | | nr | 0-10 oocyst/ 100L | | | nr | |  |
|  | |  | |  | |  | |  | | |  | |  | |  | | |  | |  | |  |  | | |  | |  |
| ***Campylobacter*** | | St. Pierre, 2009 | | Quebec, Canada, | | River and streams | | Culture + PCR assay; MPN | | nr | | | 2488 | | | 1071 | | | 21.6%- 57.8% (depending on season) | | 0.4 - <4 organisms/L – 25% sites  4- <40 organisms/L – 13% sites  ≥ 40 organisms/L- 5 % sites | | | | 0.4- 604.5 organisms/L | |  | |
|  | | Wilkes *et al.* 2009 | | Ontario, Canada | | River and streams | | Culture+ PCR assay | | | nr | | 809 | | 181 | | | 0-34% depending on season | | nr | | nr | nr | | |  | |  |
|  | | Wilkes *et al.* 2011 | | Ontario, Canada | | River and streams | | Culture+ PCR assay | | | nr | | 1171 | | 289 | | | 25% | | nr | | nr | nr | | |  | |  |
|  | | Jokinen *et al.* 2011 | | Alberta,  Canada | | River | | Culture+ PCR assay | | | nr | | 342 | | 91 | | | 27% | | nr | | nr | nr | | |  | |  |
|  | | Van Dyke *et al.* 2010 | | Ontario, Canada | | Rivers | | qPCR  Culture | | | 49-72% | | 446 | | 290 | | | 57-79% by qPCR;  0- 23% by culture | | nr | | nr | 58 cells/ L  (40-80/L) | | | 0-1300 cells/L | |  |
|  | | Khan *et al.* 2013 | | Ontario, Canada | | Beaches/ Lake  Rivers | | MPN + Culture + PCR assay | | |  | | 289  100 | | 35  14 | | | 12%  14%  (5-38% depending on location & season) | | nr  nr | | nr  nr | nr  nr | | | 3-30 cells/L | |  |
| ***E. coli* O157** | | Johnson *et al.* 2003 | | Alberta, Canada | | River | | Culture+ PCR | | | nr | | 1483 | | 13 | | | 0.9% | | nr | | nr | nr | | |  | |  |
|  | | Wilkes *et al.* 2009 | | Ontario, Canada | | River and streams | | Culture+ PCR assay | | | nr | | 823 | | 5 | | | 0-1% depending on season | | nr | | nr | nr | | |  | |  |
|  | | Wilkes et  al. 2011 | | Ontario, Canada | | River and streams | | Culture+ PCR assay | | | nr | | 1186 | | 15 | | | 1% | | nr | | nr | nr | | |  | |  |
|  | | Jokinen *et al.* 2011 | | Alberta,  Canada | | River | | Culture+ PCR assay | | | nr | | 342 | | 8 | | | 2.3% | | nr | | nr | nr | | |  | |  |

| **Norovirus**  **GI & GII** | Corsi *et al.* 2014 | USA | Milwaukee River | RT-PCR | nr | nr | nr | 10% (GI)  1.6% (GII) | 0, 4, 13 gc/L (GI)  0 gc/L (GII) | nr  nr | nr  nr | (0-400 gc/L)  0-10 gc/ L (GII) |
| --- | --- | --- | --- | --- | --- | --- | --- | --- | --- | --- | --- | --- |
|  |  |  |  |  |  |  |  |  |  |  |  |  |
| **GI & GII** | Wyn-Jones *et al.* 2011 | European countries | Recreational fresh waters | RT- PCR | nr | 928 | 58 | 6.3% | nr | nr | nr |  |
| **All norovirus** | Lodder & de Roda Husman 2005 | The Netherlands | Maas and Waal Rivers | RT-PCR | nr | 8 | 8 | 100% | 2x10^2- 2x10^3 PDU^4^/L |  |  | 95% confidence range 0.2- 4.6x 10^4 PDU^4^/L |
|  | Westrell *et al.* 2006 | The Netherlands | Meuse River | RT-PCR | nr | nr | nr | nr | 33.3 PDU^4,5^/L | nr | 0.062 PDU^4,5^/L |  |
|  |  |  |  |  |  |  |  |  |  |  |  |  |

^1^Reported values adjusted for recovery

^2^ Mean, median, standard deviations calculated from raw values published in paper

^3^Genomic equivalents

^4^PDU= PCR detection units

^5^Gamma probability function parameters α= 0.0855; λ= 370.2 (parameters that were fit to annual 2001 data)

Table S3: Small system treatment categories and estimated log-removals by pathogen for each treatment category (log removals taken from Health Canada QMRA model v13_07)

|  | **Treatment Category Details** | | | **log10-Removal Values/Ranges for Reference Pathogens** | | | | |  |
| --- | --- | --- | --- | --- | --- | --- | --- | --- | --- |
| **Cat**. | **Coagulation** | **Filtration** | **Disinfection ^a^** | ***Campy.*** | ***E. coli* O157** | **Rotavirus** | ***Crypto.*** | ***Giardia*** |  |
| 1 | Coag/floc/sed | RG(coag/sed) | Optional chemical ^b^ | 2.42 to 10.42 | 2.42 to 10.42 | 2.87 to 7.69 | 4.27 to 4.67 | 3.53 to 7.53 | |
| 2 | - | RG(direct filt) | Chemical ^b^ | 2.37 to 9.36 | 1.49 to 9.36 | 0.62 to 5.41 | 2.97 to 3.37 | 2.87 to 6.86 | |
| 3 | - | RG(no coag)/SS or bag filt. | Chemical ^b^ | 1.56 to 10.69 | 0.68 to 10.69 | 0.80 to 7.00 | 1.11 to 5.06 | 1.24 to 8.88 | |
| 4 | Coag/floc/sed | MF/UF & optional RG/SS | Chemical ^b^ | 7.16 to 20.24 | 6.28 to 20.24 | 2.89 to 12.88 | 7.99 to 13.33 | 7.80 to 17.11 | |
| 5 | Coag/floc/sed | RG(coag/sed) | Chemical ^b^ & UV | 8.43 to 15.42 | 8.05 to 15.92 | 6.98 to 11.77 | 8.70 to 9.10 | 7.54 to 11.53 | |
| 6 | - | RG(direct filt) | Chemical ^b^ & UV | 7.37 to 14.36 | 6.99 to 14.86 | 4.70 to 9.49 | 7.40 to 7.80 | 6.87 to 10.86 | |
| 7 | - | RG(no coag)/SS or bag filt. | Chemical ^b^ & UV | 6.56 to 15.69 | 6.18 to 16.19 | 4.88 to 11.08 | 5.54 to 9.49 | 5.24 to 12.88 | |
| 8 | Optional coag/floc/sed | MF/UF | Chemical ^b^ & UV | 10.61 to 22.55 | 10.23 to 23.05 | 5.21 to 14.78 | 10.56 to 13.10 | 10.19 to 16.23 | |
| 9 | - | - | Cl_2_ or ClO_2_ | 4.00 to 8.00 | 4.00 to 6.01 | 4.01 to 4.82 | 0 to 0.01 | 0.21 to 0.76 | |
| 10 | - | - | Chloramine | 1.01 | 0.13 | 0.03 | 0 | 0.01 | |
| 11 | - | - | Chemical ^b^ & UV | 6.01 to 13.00 | 5.63 to 13.50 | 4.11 to 8.90 | 4.43 to 4.83 | 4.01 to 8.00 | |
| 12 | Coag/floc/sed | - | Chemical ^b^ | 2.56 to 9.55 | 1.68 to 9.55 | 1.79 to 6.58 | 1.86 to 2.26 | 1.62 to 5.61 | |
| 13 | - | RG(no coag)/SS | - | 0.55 to 2.69 | 0.55 to 2.69 | 0.77 to 2.18 | 1.11 to 4.66 | 1.23 to 4.88 | |
| 14 | No treatment | | | 0 | 0 | 0 | 0 | 0 | |
| 15 | Optional coag/floc | MF/UF | Chemical ^b^ | 5.61 to 16.00 | 4.73 to 16.00 | 1.13 to 8.94 | 6.13 to 6.81 | 6.19 to 10.62 | |
| 16 | - | RG(no coag)/SS & MF/UF | Chemical ^b^ | 6.16 to 18.69 | 5.28 to 18.69 | 1.90 to 11.12 | 7.24 to 11.47 | 7.42 to 15.50 | |
| 17 | Coag/floc and/or sed/clar | - | Chemical ^b^ & UV | 6.01 to 14.55 | 5.63 to 15.05 | 4.11 to 10.66 | 4.43 to 6.69 | 4.01 to 9.61 | |
| 18 | - | - | Chemical ^b^ (any two) | 5.01 to 16.00 | 4.13 to 14.01 | 4.03 to 8.83 | 0 to 0.41 | 0.22 to 4.76 | |
| 19 | “Other” Treatment | | | Assumed some combination of filtration & chemical disinfection | | | | | |

^a^Assumes default values for disinfection contact time (20 min), concentration (0.20 mg/L), temperature (10^o^C), pH (6.00), and UV dose (40 mJ/cm^2^) from Health Canada model

^b^Range considers any one of free chlorine, chloramine, ozone, or chlorine dioxide

Abbreviations: SW = Surface water sources; GW = Ground water sources; RG = Rapid granular filtration; MF = microfiltration; UF = ultrafiltration; SS = slow sand filtration

Table S4. Results of sensitivity analysis: Top three factors most correlated with the number of predicted illnesses associated with the consumption of private Canadian water supplies, organised by pathogen

| **Model** | **Top 3 Factors** | **Spearman Rank**  **Correlation Coefficient** |
| --- | --- | --- |
| ***Giardia*** | 1. Concentration in groundwater (C)  2. Probability of illness given infection (P_ill/inf_)  3. Adult daily water intake (V) | 0.866  0.382  0.195 |
| ***Cryptosporidium*** | 1. Concentration in groundwater (C)  2. Probability of illness given infection (P_ill/inf_)  3. Prevalence rate (PR) | 0.828  0.326  0.267 |
| ***Campylobacter*** | 1. Probability of illness given infection (P_ill/inf_)  2. Concentration in groundwater (C)  3. Adult daily water intake (V) | 0.694  0.602  0.187 |
| ***E. coli* O157** | 1. Prevalence rate (PR)  2. Concentration in groundwater (C)  3. Probability of illness given infection (P_ill/inf_) | 0.755  0.442  0.211 |
| **Norovirus** | 1. Prevalence rate (PR)  2. Probability of illness given infection (P_ill/inf_)  3. Total Population served | 0.705  0.664  0.077 |

Table S5. Treatment distributions and corresponding populations for each treatment category, pathogen, and water source

| **Treatment Category**  **(Types Included)^1^** | **Treatment log-Reduction Distributions^2^** | | | **Population at Risk^3^** | |
| --- | --- | --- | --- | --- | --- |
|  | **Pathogen** | **GW Systems** | **SW Systems** | **GW Systems** | **SW Systems** |
| No treatment  (14) | - | - | - | (107,142;  108,885) | (6,017;  6,115) |
| Membrane filtration^4^  (4, 15, 16) | *Giardia*  *Cryptosporidium*  *Campylobacter*  *E. coli* O157  Rotavirus^8^ | (6.19, 7.56, 15.50)  (6.13, 6.13, 11.47)  (5.61, 8.00, 18.69)  (4.73, 7.25, 18.69)  (1.13, 3.17, 11.12) | (6.19, 7.24, 17.11)  (6.13, 6.13, 13.33)  (5.61, 7.84, 20.24)  (4.73, 7.08, 20.24)  (1.13, 2.97, 12.88) | (45,581;  46,323) | (45,829;  46,574) |
| Media filtration^5^  (1, 2, 3, 13, 19) | *Giardia*  *Cryptosporidium*  *Campylobacter*  *E. coli* O157  Rotavirus^8^ | (1.23, 3.21, 8.88)  (1.11, 2.46, 5.06)  (0.55, 4.89, 10.69)  (0.55, 3.13, 10.69)  (0.62, 2.58, 7.69) | (1.24, 4.68, 8.88)  (1.11, 3.52, 5.06)  (1.56, 4.86, 10.69)  (0.68, 4.56, 10.69)  (0.62, 3.43, 7.69) | (165,353;  168,042) | (159,492;  162,085) |
| Chemical disinfection^6^  (9, 10, 12, 18) | *Giardia*  *Cryptosporidium*  *Campylobacter*  *E. coli* O157  Rotavirus^8^ | (0.01, 0.01, 5.61)  (0.00, 0.00, 2.26)  (1.01, 2.69, 16.00)  (0.13, 1.85, 14.01)  (0.03, 1.86, 8.83) | (0.01, 0.01, 5.61)  (0.00, 0.00, 2.26)  (1.01, 3.55, 16.00)  (0.13, 2.67, 14.01)  (0.03, 2.48, 8.83) | (481,331;  489,157) | (196,121;  199,310) |
| UV & chemical disinfection^7^  (5, 6, 7, 8, 11, 17) | *Giardia*  *Cryptosporidium*  *Campylobacter*  *E. coli* O157  Rotavirus^8^ | (4.01, 5.24, 16.23)  (4.43, 4.43, 13.10)  (6.01, 7.86, 22.55)  (5.63, 7.66, 23.05)  (4.11, 5.61, 14.78) | (4.01, 6.05, 16.23)  (4.43, 4.43, 13.10)  (6.01, 8.79, 22.55)  (5.63, 8.63, 23.05)  (4.11, 6.28, 14.78) | (20,941;  21,281) | (63,897;  64,936) |

^1^See Table S3 (supplementary material) for details on specific treatment types

^2^PERT distribution (minimum, most likely, maximum)

^3^Uniform distribution (minimum, maximum) accounting for bottled water use and possible home treatment for all systems serving < 1000 people

^4^Membrane filtration (micro or ultra-filtration) with chemical disinfection, with/without other treatment (no UV disinfection)

^5^Media filtration with.without other treatment (no UV or membrane filtration)

^6^Chemical disinfection (ozone, chlorine, chlorine dioxide, chloramines) with/without coagulation/ flocculation/ sedimentation

^7^UV disinfection and chemical disinfection, with/without other treatment

^8^Rotavirus log-reductions used as surrogate for norovirus log-reductions

Table S6. Multi-pathogen QMRA results for private well supplies in Canada

|  | **Mean Daily  Prob. of Infection^1^ (90% Prob. Interval)** | **Mean Annual Prob. of Infection^2^** | **Mean Projected Infections^3^** | **Mean Projected Symptomatic Illnesses**  **(90% Prob. Interval)** | **% of Total**  **AGI Illnesses^4^** |
| --- | --- | --- | --- | --- | --- |
| ***Giardia*** | 5.30E-04  (3.19E-07; 1.57E-03) | 9.36E-04 | 2,680 | 1,207  (2; 7,136) | 1.55% |
| ***Cryptosporidium*** | 1.45E-03  (1.57E-05; 5.62E-03) | 8.88E-03 | 25,414 | 11,398  (238; 45,141) | 14.6% |
| ***Campylobacter*** | 6.59E-03  (3.07E-04; 2.08E-02) | 9.24E-03 | 26,443 | 9,273  (1,180; 19,980) | 11.9% |
| ***E. coli* O157** | 8.24E-05  (1.79E-05; 1.84E-04) | 5.58E-04 | 1,595 | 637  (124; 1,528) | 0.82% |
| **Norovirus** | 4.39E-01  (9.73E-02; 5.38E-01) | 3.53E-02 | 100,975 | 55,558  (24,323; 95,709) | 71.2% |
| **Total projected cases of *Giardia*, *Cryptosporidium*, *Campylobacter*,**  ***E. coli* O157 and norovirus** | | | | 78,073  (38,466; 128,109) |  |

^1^Daily probability of infection for a person using a contaminated private well (excludes prevalence rate)

^2^Annual probability of infection for any person using a private well (includes prevalence rate)

^3^Susceptible population is approximately 2,861,602

^4^Mean projected symptomatic illnesses divided by 78,073

Table S7. Multi-pathogen QMRA results for small Canadian groundwater systems serving <1000 people

|  | **Treatment Category** | **Mean Daily  Prob. of Infection^1^ (90% Prob. Interval)** | **Mean Annual Prob. of Infection^2^** | **Mean Projected Infections^3^** | **Mean Projected Symptomatic Illnesses**  **(90% Prob. Interval)** | **% of Total**  **AGI Illnesses^4^** |
| --- | --- | --- | --- | --- | --- | --- |
| ***Giardia*** | No Treatment | 5.30E-04  (3.19E-07; 1.57E-03) | 9.36E-04 | 101 | 46 (0; 268) |  |
|  | Membrane Filtration | 2.16E-11  (0; 3.96E-11) | 1.06E-10 | 0 | 0 |  |
|  | Media  Filtration | 1.12E-06  (2.89E-12; 1.88E-06) | 5.29E-06 | 1 | 0 (0; 1) |  |
|  | Chemical Disinfection | 1.61E-04  (1.14E-08; 3.75E-04) | 3.43E-04 | 166 | 75 (0; 364) |  |
|  | UV & Chemical Disinfection | 3.33E-09  (0; 5.46E-09) | 1.63E-08 | 0 | 0 |  |
|  | **Total projected number of symptomatic illnesses (*Giardia*)** | | | | 121 (0; 619) | 0.93% |
| ***Cryptosporidium*** | No Treatment | 1.45E-03  (1.57E-05; 5.62E-03) | 8.88E-03 | 959 | 430 (9; 1,702) |  |
|  | Membrane Filtration | 3.44E-10  (3.33E-13; 1.22E-09) | 5.16E-09 | 0 | 0 |  |
|  | Media  Filtration | 9.40E-06  (9.98E-09; 3.39E-05) | 1.28E-04 | 21 | 10 (0; 35) |  |
|  | Chemical Disinfection | 7.63E-04  (5.15E-06; 2.84E-03) | 5.51E-03 | 2,674 | 1,199 (14; 5,459) |  |
|  | UV & Chemical Disinfection | 1.11E-08  (7.50E-13; 4.37E-08) | 1.68E-07 | 0 | 0 |  |
|  | **Total projected number of symptomatic illnesses (*Cryptosporidium*)** | | | | 1639 (27; 7,108) | 12.6% |
|  | **Treatment Category** | **Mean Daily  Prob. of Infection (90% Prob. Interval)** | **Mean Annual Prob. of Infection** | **Mean Projected Infections** | **Mean Projected Symptomatic Illnesses**  **(90% Prob. Interval)** | **% of Total AGI Illnesses** |
| ***Campylobacter*** | No Treatment | 6.59E-03  (3.07E-04; 2.07E-02) | 9.24E-03 | 998 | 350 (45; 753) |  |
|  | Membrane Filtration | 5.49E-10  (0; 2.57E-09) | 2.73E-09 | 0 | 0 |  |
|  | Media  Filtration | 1.36E-05  (1.22E-11; 4.16E-05) | 5.87E-05 | 10 | 3 (0; 12) |  |
|  | Chemical Disinfection | 3.37E-05  (1.46E-12; 1.75E-04) | 1.51E-04 | 73 | 25 (0; 137) |  |
|  | UV & Chemical Disinfection | 3.07E-10  (0; 1.61E-09) | 1.54E-09 | 0 | 0 |  |
|  | **Total projected number of symptomatic illnesses (*Campylobacter*)** | | | | 378 (45; 818) | 2.90% |
| ***E. coli* O157** | No Treatment | 8.24E-05  (1.79E-05; 1.84E-04) | 5.58E-04 | 60 | 24 (5; 58) |  |
|  | Membrane Filtration | 4.58E-11  (0; 2.47E-10) | 3.18E-10 | 0 | 0 |  |
|  | Media  Filtration | 6.44E-07  (3.26E-12; 3.27E-06) | 4.46E-06 | 1 | 0 (0; 1) |  |
|  | Chemical Disinfection | 2.99E-06  (7.59E-13; 1.72E-05) | 2.06E-05 | 10 | 4 (0; 23) |  |
|  | UV & Chemical Disinfection | 7.47E-12  (0; 4.35E-11) | 5.18E-11 | 0 | 0 |  |
|  | **Total projected number of symptomatic illnesses (*E. coli* O157)** | | | | 28 (5; 72) | 0.21% |
| **Norovirus** | No Treatment | 4.39E-01  (9.73E-02; 5.38E-01) | 3.53E-02 | 3,812 | 2,098 (917; 3,616) |  |
|  | Membrane Filtration | 2.76E-02  (1.21E-07; 1.80E-01) | 1.45E-02 | 665 | 365 (0; 1,249) |  |
|  | Media  Filtration | 6.15E-02  (7.98E-06; 3.78E-01) | 2.16E-02 | 3,597 | 1,979 (8; 4,946) |  |
|  | Chemical Disinfection | 1.10E-01  (6.39E-06; 4.74E-01) | 2.41E-02 | 11,671 | 6,417 (20; 14,903) |  |
|  | UV & Chemical Disinfection | 9.08E-05  (1.35E-10; 4.13E-04) | 8.79E-04 | 19 | 10 (0; 52) |  |
|  | **Total projected number of symptomatic illnesses (norovirus)** | | | | 10,869 (2,211; 22,736) | 83.4% |
| **Total projected cases of *Giardia*, *Cryptosporidium*, *Campylobacter*,**  ***E. coli* O157 and norovirus** | | | | | 13,035 (3,416, 25,698) |  |

^1^Daily probability of infection for a person using a contaminated small groundwater system (excludes prevalence rate)

^2^Annual probability of infection for any person using a small groundwater system (includes prevalence rate)

^3^Susceptible populations are approximately 108,014 (no treatment), 49,952 (membrane filtration), 166,698 (media filtration), 485,244 (chemical disinfection), and 21,111 (UV and chemical disinfection)

^4^Mean projected symptomatic illnesses (summed for all treatment categories) divided by 13,035

Table S8. Multi-pathogen QMRA results for small Canadian surface water systems serving <1000 people

|  | **Treatment Category** | **Mean Daily  Prob. of Infection^1^ (90% Prob. Interval)** | **Mean Annual Prob. of Infection^2^** | **Mean Projected Infections^3^** | **Mean Projected Symptomatic Illnesses**  **(90% Prob. Interval)** | **% of Total**  **AGI Illnesses^4^** |
| --- | --- | --- | --- | --- | --- | --- |
| ***Giardia*** | No Treatment | 4.26E-03  (8.92E-06; 1.51E-02) | 5.54E-02 | 336 | 151 (1, 611) |  |
|  | Membrane Filtration | 2.83E-10  (0; 4.81E-10) | 2.21E-08 | 0 | 0 |  |
|  | Media  Filtration | 2.51E-06  (7.62E-12; 4.27E-06) | 1.73E-04 | 28 | 12 (0; 21) |  |
|  | Chemical Disinfection | 1.23E-03  (2.81E-07; 3.81E-03) | 2.37E-02 | 4,690 | 2,124 (1; 11,556) |  |
|  | UV & Chemical Disinfection | 1.74E-08  (0; 3.18E-08) | 1.08E-06 | 0 | 0 |  |
|  | **Total projected number of symptomatic illnesses (*Giardia*)** | | | | 2,288 (6; 12,120) | 18.9% |
| ***Cryptosporidium*** | No Treatment | 9.38E-05  (6.34E-07; 3.28E-04) | 6.02E-03 | 37 | 17 (0; 73) |  |
|  | Membrane Filtration | 1.61E-11  (2.60E-15; 5.78E-11) | 1.34E-09 | 0 | 0 |  |
|  | Media  Filtration | 1.54E-07  (9.91E-11; 4.40E-07) | 1.29E-05 | 2 | 1 (0; 3) |  |
|  | Chemical Disinfection | 4.81E-05  (1.93E-07; 1.72E-04) | 3.34E-03 | 660 | 300 (1; 1,230) |  |
|  | UV & Chemical Disinfection | 6.68E-10  (3.90E-14; 2.29E-09) | 5.50E-08 | 0 | 0 |  |
|  | **Total projected number of symptomatic illnesses (*Cryptosporidium*)** | | | | 317 (1; 1,310) | 2.62% |
|  | **Treatment Category** | **Mean Daily  Prob. of Infection (90% Prob. Interval)** | **Mean Annual Prob. of Infection** | **Mean Projected Infections** | **Mean Projected Symptomatic Illnesses**  **(90% Prob. Interval)** | **% of Total AGI Illnesses** |
| ***Campylobacter*** | No Treatment | 1.48E-02  (6.11E-06; 8.41E-02) | 1.08E-01 | 656 | 232 (1; 868) |  |
|  | Membrane Filtration | 1.15E-08  (0; 2.70E-09) | 1.76E-06 | 0 | 0 |  |
|  | Media  Filtration | 2.24E-05  (1.08E-12; 1.28E-05) | 1.03E-03 | 166 | 59 (0;58) |  |
|  | Chemical Disinfection | 9.59E-05  (3.00E-14; 7.89E-05) | 3.09E-03 | 610 | 222 (0; 417) |  |
|  | UV & Chemical Disinfection | 2.14E-09  (0; 5.98E-10) | 1.78E-07 | 0 | 0 |  |
|  | **Total projected number of symptomatic illnesses (*Campylobacter*)** | | | | 513 (1; 1,433) | 4.23% |
| ***E. coli* O157** | No Treatment | 8.22E-05  (1.80E-05; 1.85E-04) | 3.07E-04 | 2 | 1 (0; 2) |  |
|  | Membrane Filtration | 5.02E-11  (0; 2.83E-10) | 1.94E-10 | 0 | 0 |  |
|  | Media  Filtration | 1.78E-07  (4.02E-13; 7.07E-07) | 6.69E-07 | 0 | 0 |  |
|  | Chemical Disinfection | 1.85E-06  (1.69E-13; 9.66E-06) | 6.85E-06 | 1 | 1 (0; 3) |  |
|  | UV & Chemical Disinfection | 4.15E-12  (0; 2.11E-11) | 1.55E-11 | 0 | 0 |  |
|  | **Total projected number of symptomatic illnesses (*E. coli* O157)** | | | | 1 (0; 4) | 0.008% |
| **Norovirus** | No Treatment | 5.15E-01  (4.69E-01; 5.46E-01) | 5.87E-02 | 356 | 196 (76; 344) |  |
|  | Membrane Filtration | 5.74E-01  (2.18E-07; 3.61E-01) | 3.10E-02 | 1,432 | 788 (0; 2,285) |  |
|  | Media  Filtration | 6.36E-02  (2.16E-05; 3.79E-01) | 3.73E-02 | 5,998 | 3,298 (37; 8,172) |  |
|  | Chemical Disinfection | 1.31E-01  (2.21E-05; 4.86E-01) | 4.29E-02 | 8,473 | 4,648 (43; 10,453) |  |
|  | UV & Chemical Disinfection | 1.28E-04  (3.79E-10; 6.90E-04) | 2.07E-03 | 133 | 73 (0; 412) |  |
|  | **Total projected number of symptomatic illnesses (norovirus)** | | | | 9,003 (1,790; 18,930) | 74.3% |
| **Total projected cases of *Giardia*, *Cryptosporidium*, *Campylobacter*,**  ***E. coli* O157 and norovirus** | | | | | 12,122 (2,974; 26,274) |  |

^1^Daily probability of infection for a person using a contaminated small surface water system (excludes prevalence rate)

^2^Annual probability of infection for any person using a small surface water system (includes prevalence rate)

^3^Susceptible populations are approximately 6,066 (no treatment), 46,202 (membrane filtration), 160,789 (media filtration), 197,716 (chemical disinfection), and 64,417 (UV and chemical disinfection)

^4^Mean projected symptomatic illnesses (summed for all treatment categories) divided by 12,122
